# Complete Genome Sequence of an *N*-Acyl Homoserine Lactone Producer, *Breoghania* sp. Strain L-A4, Isolated from Rhizosphere of *Phragmites australis* in a Coastal Wetland

**DOI:** 10.1128/MRA.01539-18

**Published:** 2019-01-31

**Authors:** Changfei He, Li Zheng, Jinfeng Ding, Wei Gao, Wendan Chi, Yu Ding

**Affiliations:** a Key Laboratory for Marine Bioactive Substances and Modern Analytical Technology, First Institute of Oceanography, Ministry of Natural Resources, Qingdao, China; b Laboratory for Marine Ecology and Environmental Science, Qingdao National Laboratory for Marine Science and Technology, Qingdao, China; University of Rochester School of Medicine and Dentistry

## Abstract

The *Breoghania* sp. strain L-A4 was isolated from the rhizosphere of *Phragmites australis* in the Qinhaungdao coastal wetland in China. Here, we present the complete genome sequence of strain L-A4, which consists of a chromosome of 5,029,620 bp with a G+C content of 64.53% and 4,964 coding DNA sequences. This strain was the first detected to produce *N*-acyl homoserine lactone (AHL) signals in a member of this genus.

## Abstract (precis)

The *Breoghania* sp. strain L-A4 was isolated from the rhizosphere of *Phragmites australis* in the Qinhaungdao coastal wetland in China.

## ANNOUNCEMENT

Rhizobacteria associated with plants play an important role in the coastal wetland ecosystem. As the signal molecules of the quorum sensing (QS) system, *N*-acyl homoserine lactone (AHL) signals have been described in many plant-associated bacteria, including rhizobacteria (1). Quorum sensing is a function of microorganisms for intercellular communication which is typically used when microbial group activities are beneficial (2). The ability to produce of a wide range of antibiotics and cell wall lytic enzymes, rhizosphere competence and viability on plant surfaces, the ability to promote plant growth, and induction of plant resistance to pathogens are among the beneficial traits of rhizobacteria which are regulated by the QS system (3).

*Breoghania* belongs to the family *Cohaesibacteraceae* in the phylum *Alphaproteobacteria*, which was first described by Hwang (4, 5). Many of these species are isolated from rhizosphere soil. This strain was isolated by M8 medium plates containing 1.5% agar (6). The plates were incubated at 30°C for 48 to 72 h. Unique colonies of L-A4 were obtained.

**FIG 1:**
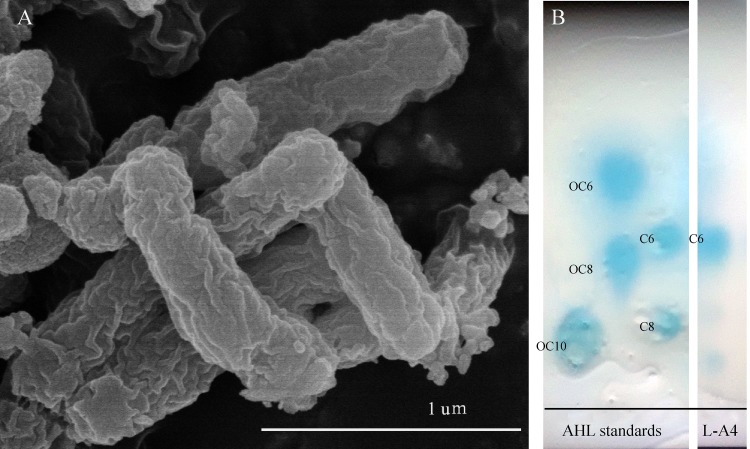
Characterization of *Breoghania* sp. strain L-A4. (A) Scanning electron microscope image of *Breoghania* sp. strain L-A4. (B) Analysis of AHLs from supernatant extracts on a reverse-phase thin-layer chromatographic (TLC) plate developed with the biosensor A. tumefaciens KYC55. C6, *N*-hexanoyl-l-homoserine lactone; OC6, *N*-(β-ketocaproyl)-l-homoserine lactone; C8, *N*-octanoyl-l-homoserine lactone; OC8, *N*-(3-oxoctanoyl)-l-homoserine; OC10, *N*-(3-oxodecanoyl)-l-homoserine.

*Breoghania* sp. strain L-A4 is a long rod-shaped aerobic bacterium, approximately 0.2 to 0.4 μm wide and 0.8 to 1.2 μm long (Fig. 1A). One AHL signal, *N*-hexanoyl-l-homoserine lactone (C_6_), can be produced in *Breoghania* sp. strain L-A4 and was detected with the thin-layer chromatography (TLC) overlay method using the Agrobacterium tumefaciens KYC55 biosensor method (7, 8) (Fig. 1B). We performed complete genome sequencing of L-A4 and hope to confirm the ecological functions regulated by the QS system.

Genomic DNA of L-A4 was extracted with the PowerSoil DNA isolation kit (Mo Bio, Carlsbad, CA) following the manufacturer’s instructions. The DNA sample was broken, using a Covaris g-TUBE, into the desired size fragments (10 kb) of the library. After DNA damage repair, the hairpin-type adaptor was ligated to both ends of the DNA fragment using DNA adhesive enzyme. The 10-kb SMRTbell library was constructed with purification DNA fragments of AMPure PB magnetic beads (9). The constructed library was quantified by Qubit concentration, and the insert size was detected using an Agilent 2100 instrument (10). PacBio sequencing was performed at the Beijing Novogene Bioinformatics Technology Co., Ltd. Raw data were filtered to obtain clean data (read length, >500 bp; read quality, >0.75). A total of 35,854 reads with an average length of 14,302 bp were obtained for a total of 512,801,384 bases of sequence. *De novo* assembly was done using the Hierarchical Genome Assembly Process (HGAP) workflow implemented in the single-molecule real-time (SMRT) analysis software SMRT Link version 5.0.1 (11, 12). Genome sequences were annotated and analyzed using GeneMarkS version 4.17 (http://topaz.gatech.edu/GeneMark/) (13). tRNA operons were assessed via tRNAscan-SE version 1.3.1 (14), and rRNA operons were assessed by RNAmmer version 1.2 (15).

The genome sequence of L-A4 was assembled into one contiguous scaffold with 150-fold coverage, which represents a single circular 5,029,620-bp chromosome with a G+C content of 64.53%. The genome is predicted to contain 4,964 coding sequences. The numbers of tRNAs and rRNA operons are 49 and 6, respectively. From the chromosome of L-A4, one cluster of *luxI* and *luxR* homologues was identified, which located the genes Chr1:3738389:3739039 and Chr1:3739133:3739858, respectively. Other metabolic pathways also exist in L-A4, such as carbon metabolism and nitrogen metabolism.

### Data availability.

The complete genome sequence of *Breoghania* sp. strain L-A4 has been deposited at DDBJ/EMBL/GenBank under the accession number CP031841 (BioProject number PRJNA487520). The raw sequencing reads have been submitted to the Sequence Read Archive (SRA) under accession number SRR8101942.
